# Supplementary figures and images for: PTEX helps efficiently traffic haemoglobinases to the food vacuole in Plasmodium falciparum
Source: PLoS Pathog. 2023 Jul 31;19(7):e1011006. doi: 10.1371/journal.ppat.1011006 (PMC10414648; doi:10.1371/journal.ppat.1011006)

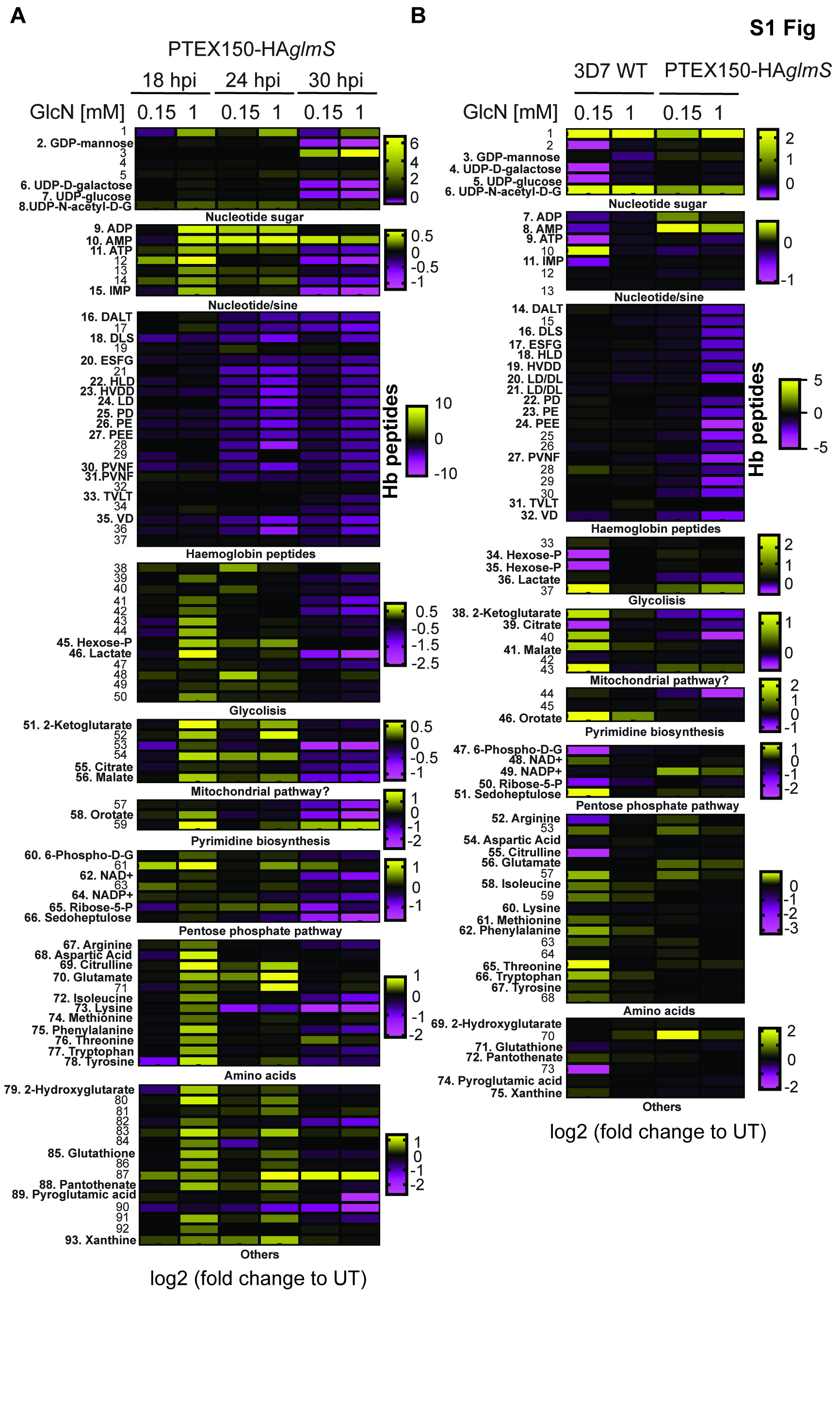

Supplement: S1 Fig — (A) Heat map corresponding to S1 Table for PTEX150-HAglmS metabolomics time course (18, 24, 30-hpi) experiment. (B) Heat map corresponding to S1 Table for 3D7 and PTEX150-HAglmS metabolomics experiment. Numbers represent metabolites listed in S1 Table, where Hb peptides have been highlighted in bold. Fold change for both heat maps was calculated using the following formula, Fold change = log2 (GlcN treated / untreated). (TIF) [file ppat.1011006.s001.tif]

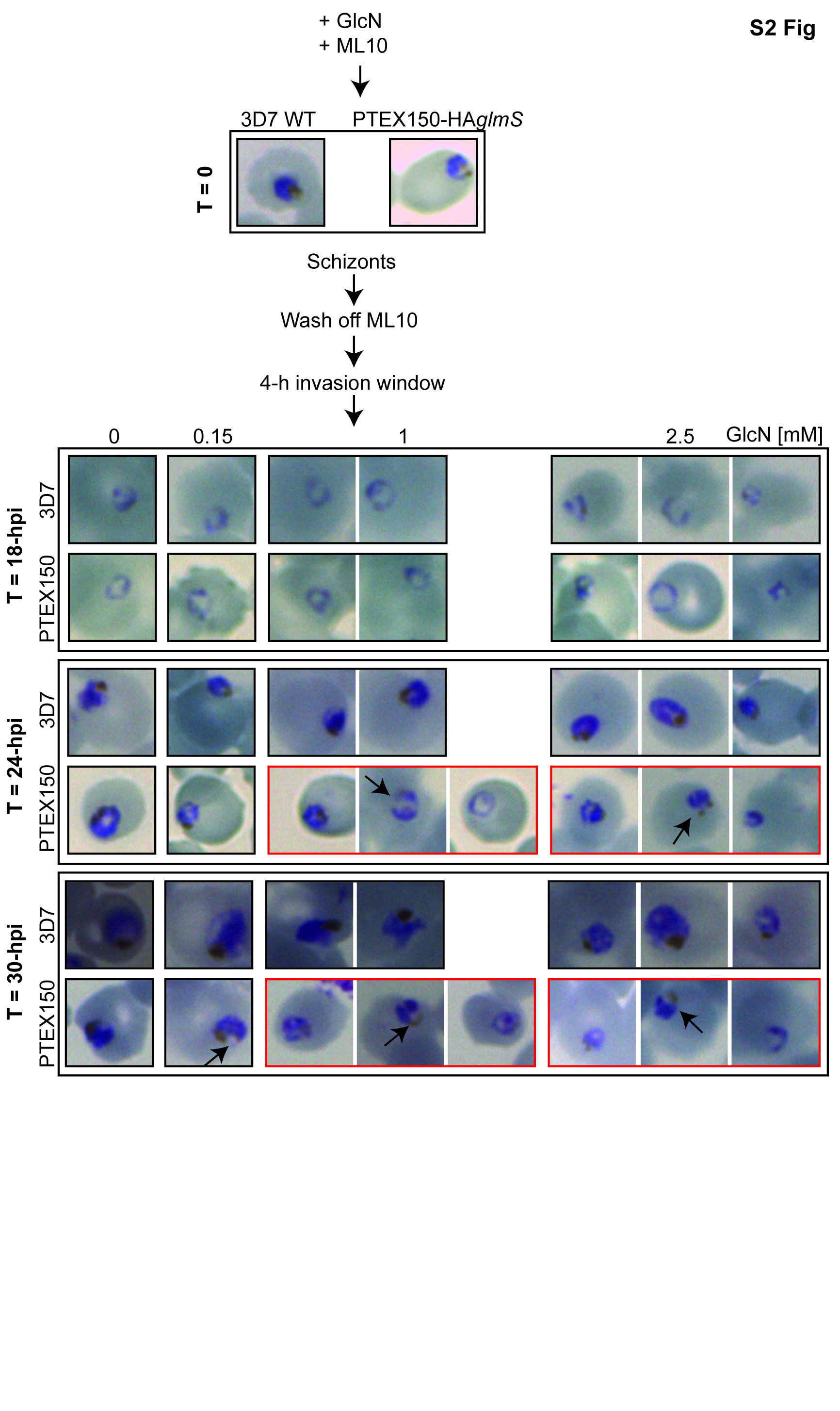

Supplement: S2 Fig — 3D7 WT and PTEX150-HAglmS parasites were treated with increasing concentration of GlcN at trophozoite stage (T = 0). Parasites were given 4-h invasion window and then blood smears were taken at 18-hpi, 24-hpi and 30-hpi. Figures are representative images from 3 biological replicates, where red box around images in 24-hpi and 30-hpi emphasize growth stall observed, this was more prominent in 2.5 mM GlcN treatment. Arrows point to expansion of the food vacuole, which was sometimes observed. (TIF) [file ppat.1011006.s002.tif]

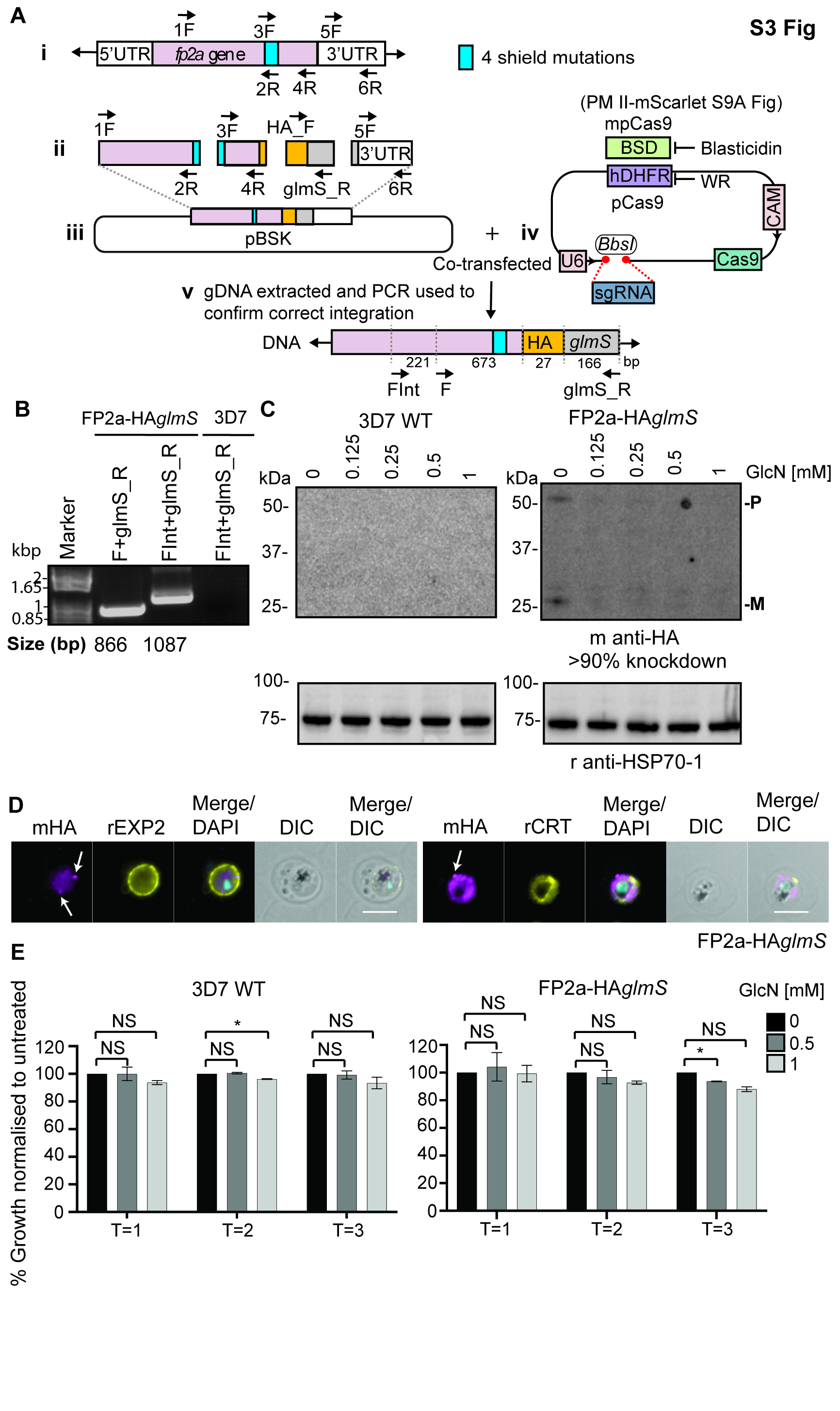

Supplement: S3 Fig — (A) i) CRISPR-Cas9 was used to append the fp2a gene with a HA tag and a glmS riboswitch. ii) This was done in a multi-step PCR, where primer 1F (forward) was located ~500 bases upstream from the gene stop codon and primer 2R (reverse) started at the gRNA cut site, where 4 shield mutations were added to the end of the primer. Primer 3F overlapped with primer 2R and had the 4 shield mutations at the start of the primer and 4R was located just before the stop codon and included the first few bases of the HA tag. The HAglmS tag was amplified using the HA_F and glmS_R primers. The 3’UTR was amplified using 5F primer, which had the last few bases of glmS at the start and the 6R primer started ~500 bp downstream from the stop codon. These 4 PCRs were then joined together. iii) The flank was ligated into the pBSK vector and transfected together with the iv) Cas9 plasmid containing the gRNA. v) When transgenic parasites were obtained, genomic DNA was extracted and PCR completed to confirm correct integration of the HAglmS tag into the target gene, where F_Int was located upstream of F1. (B) PCR was used to confirm the correct integration of the tag to the target gene, where 3D7 WT was used as a negative control for integration. The position of PCR primers used to confirm integration are shown in panel A, where FInt, is the forward PCR integration primer. F, forward primer. glmS_R, reverse primer. (C) Correct size and level of FP2a knockdown was confirmed using western blot. >90% of FP2a was knocked down after one cell cycle of GlcN treatment. P stands for pro FP2a and M stands for mature FP2a. The HA-tag adds approximately 3 kDa to the target protein. The mark on the right-hand side of the blot is an artefact. Mouse anti-HA detects FP2a-HA and rabbit anti-HSP70-1 was used as a loading control. Blot is representative of 3 biological replicates. Full-length blots are shown in S16 Fig. (D) Immunofluorescence assays where mouse anti-HA detects target protein and rabbit anti-EXP2 [file ppat.1011006.s003.tif]

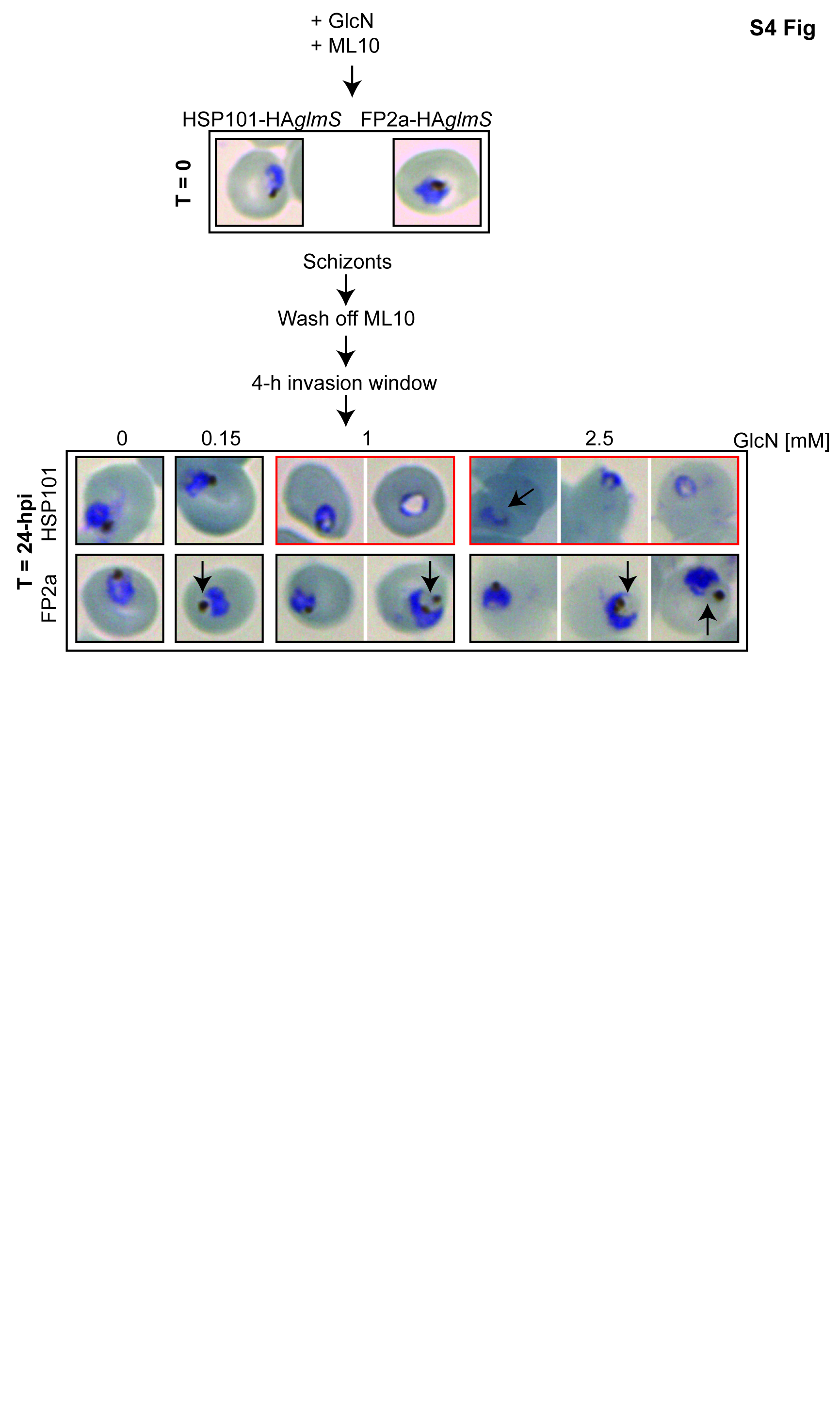

Supplement: S4 Fig — HSP101-HAglmS and FP2a-HAglmS parasites were treated with increasing concentration of GlcN at trophozoite stage (T = 0). Parasites were given 4-h invasion window and then blood smears were taken at 24-hpi. Figures are representative images from 3 biological replicates, where red box around images emphasize growth stall observed, this was more prominent in 2.5 mM GlcN treatment. Arrows point to expansion of the food vacuole, which was sometimes observed. (TIF) [file ppat.1011006.s004.tif]

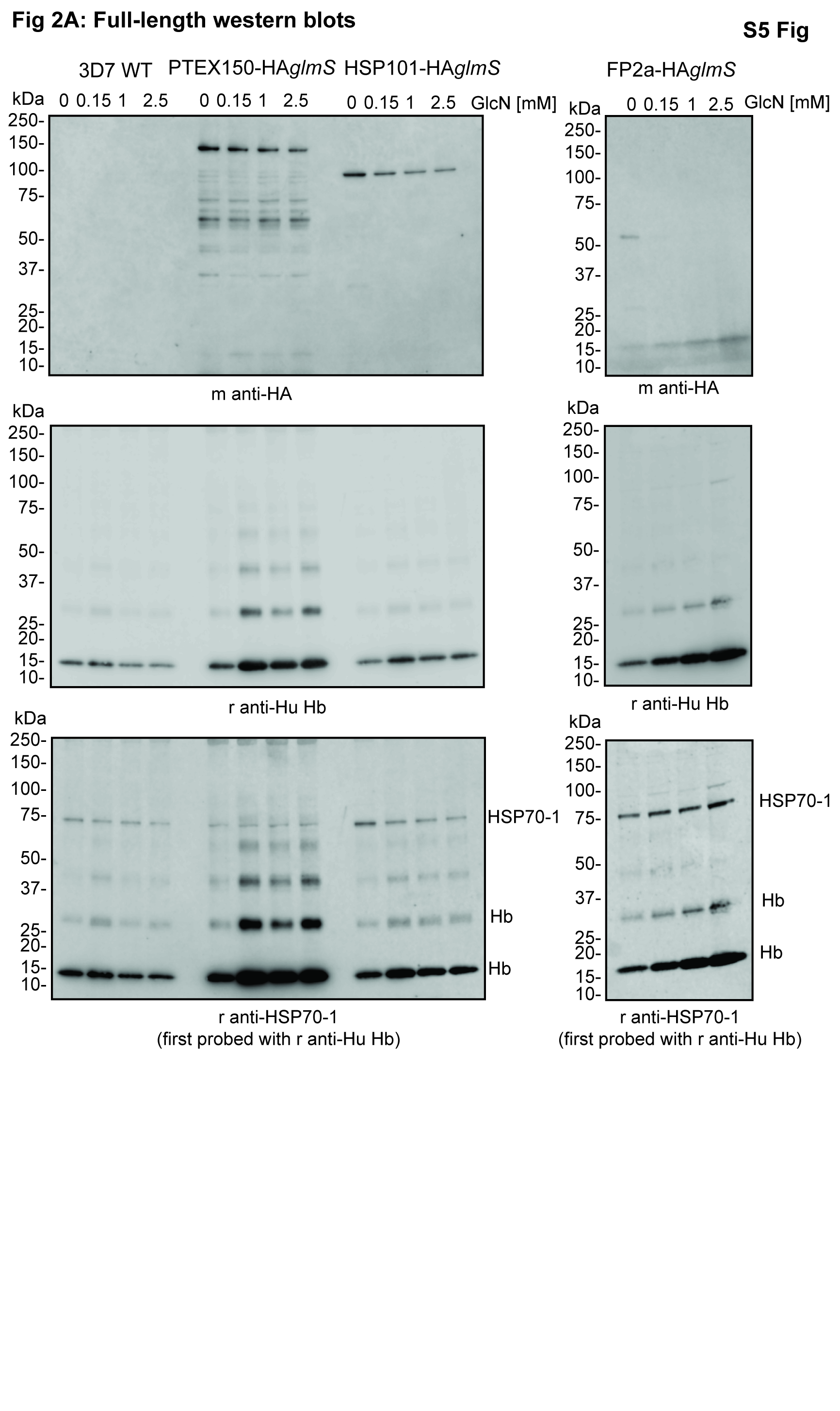

Supplement: S5 Fig — (TIF) [file ppat.1011006.s005.tif]

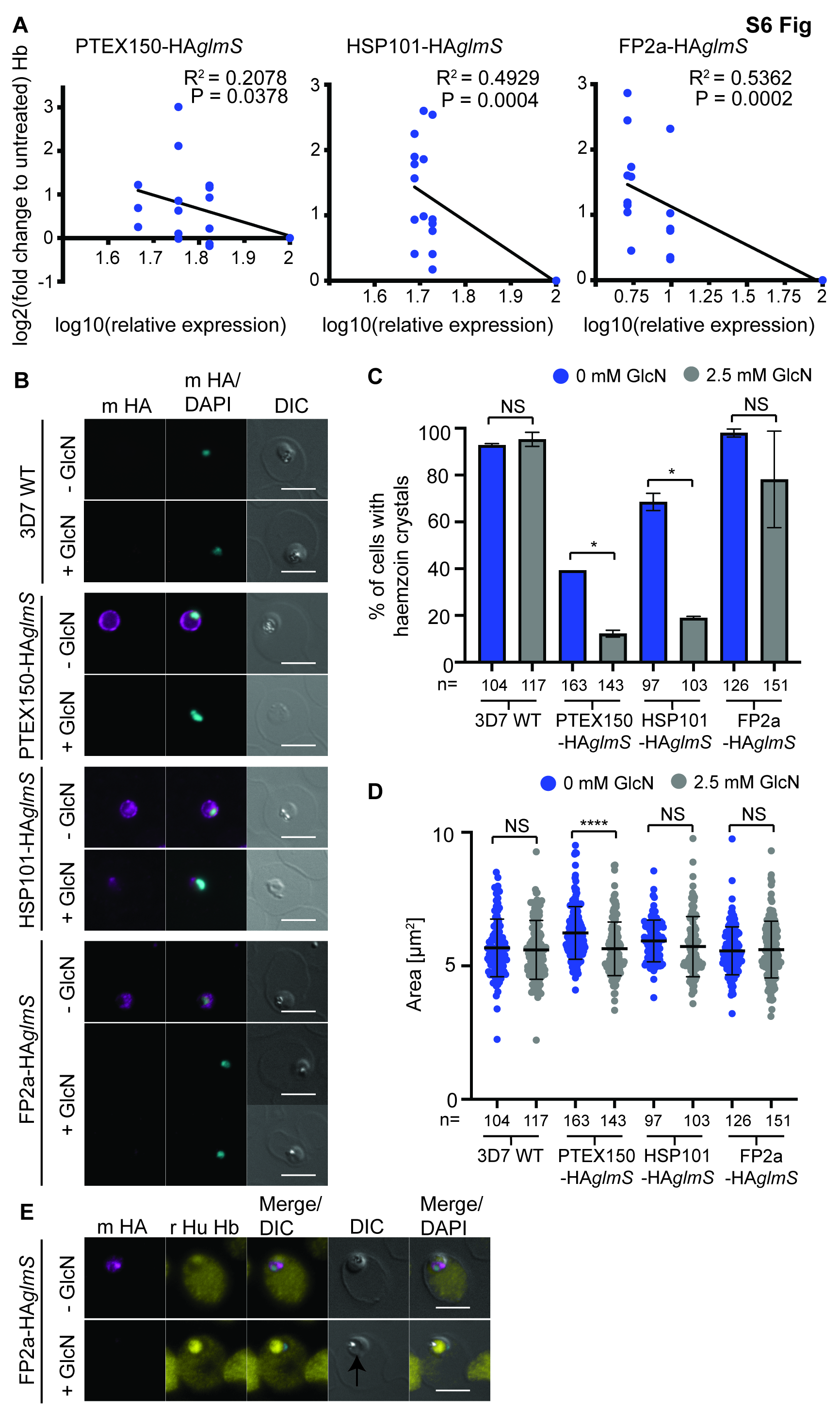

Supplement: S6 Fig — (A) Simple linear regression analysis was performed on protein expression and Hb build-up from western blots presented in Fig 2B for PTEX150-HAglmS, HSP101-HAglmS and FP2a-HAglmS. All parasite lines showed significant regression slope, where P values are shown in each graph along with R2. The blue dots on the x-axis are log10 of mean of the fold difference in protein expression for 6 biological replicates for 0.15 and 1 mM GlcN and 3 biological replicates for 2.5 mM GlcN plotted against log2 of the fold difference for individual biological replicates for Hb build-up (y-axis). The SD for the x-axis shown is as follows, PTEX150-HAglmS (X = 2, SD = 0; X = 1.82, SD = 0.06, X = 1.75, SD = 0.12, X = 1.67, SD = 0.21), HSP101-HAglmS (X = 2, SD = 0; X = 1.73, SD = 0.14, X = 1.69, SD = 0.08, X = 1.71, SD = 0.21), FP2a-HAglmS (X = 2, SD = 0; X = 0.99, SD = 0.19; X = 0.71, SD = 0.52, X = 0.74, SD = 0.61). (B) Highly-synchronous 3D7 WT, PTEX150-HAglmS, HSP101-HAglmS and FP2a-HAglmS trophozoite stage parasites were treated ± 2.5 mM GlcN for one cell cycle and harvested for IFA. Haemozoin crystals in the DIC channel were counted (present or absent). Images are representative of 3 (3D7 WT, PTEX150-HAglmS, FP2a-HAglmS) or 2 (HSP101-HAglmS) biological replicates. (C) Both PTEX150-HAglmS and HSP101-HAglmS knockdown experiments shown in panel B resulted in significantly less crystal formation compared to untreated cells when using Student’s t test with Welch correction. No significant difference in haemozoin crystal count was observed for 3D7 WT or FP2a-HAglmS parasites, although FP2a-HAglmS parasites often appeared to have smaller crystals. (*) Indicates P = 0.0247 (PTEX150-HAglmS) and P = 0.0277 (HSP101-HAglmS). Error bars = SD from 2 individuals counting. (D) Area of the parasites analysed in panel C (completed as described in Fig 2E) showed significant difference in size for GlcN treated PTEX150-HAglmS parasites compared with untreated indicating parasite growth was affected but no [file ppat.1011006.s006.tif]

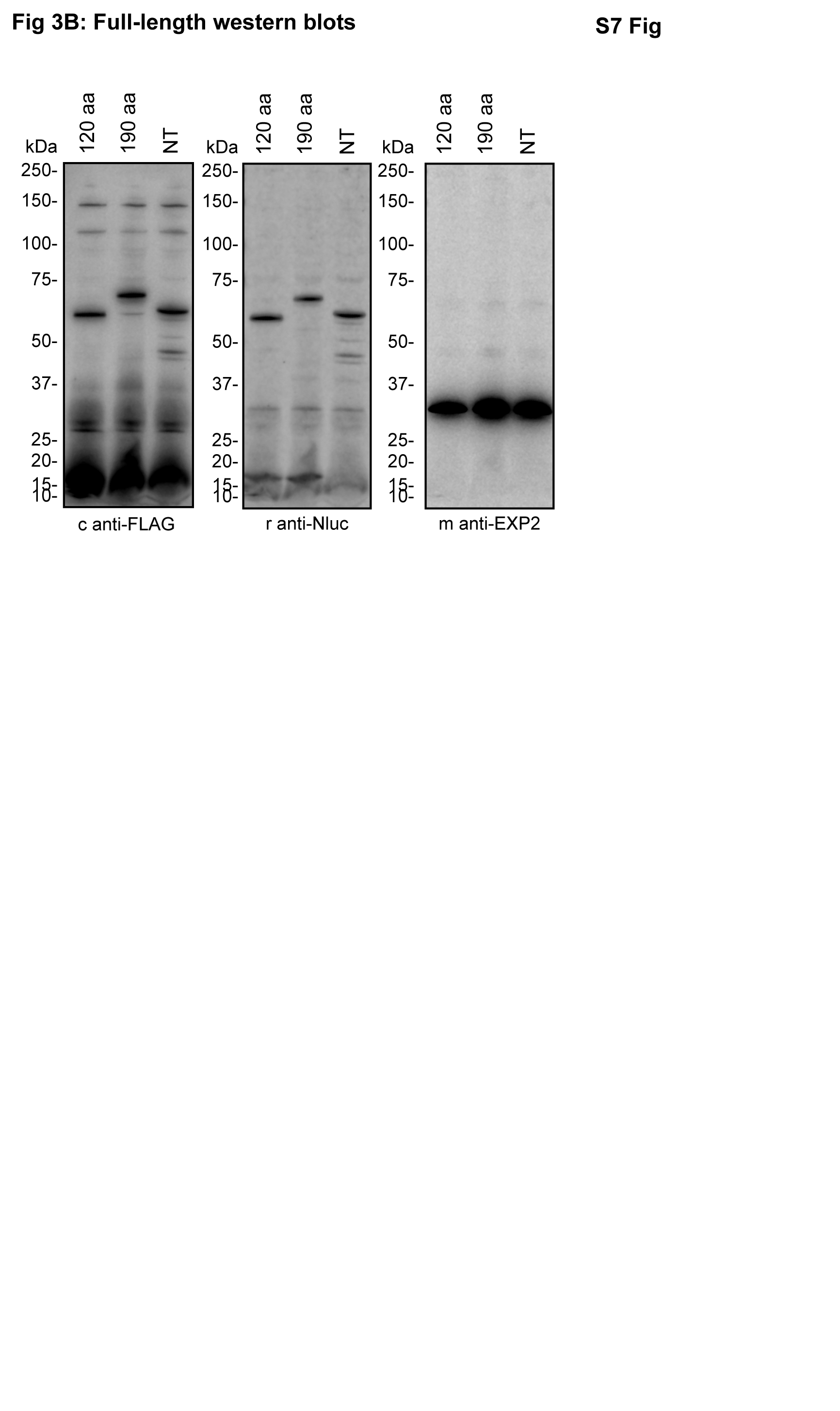

Supplement: S7 Fig — (TIF) [file ppat.1011006.s007.tif]

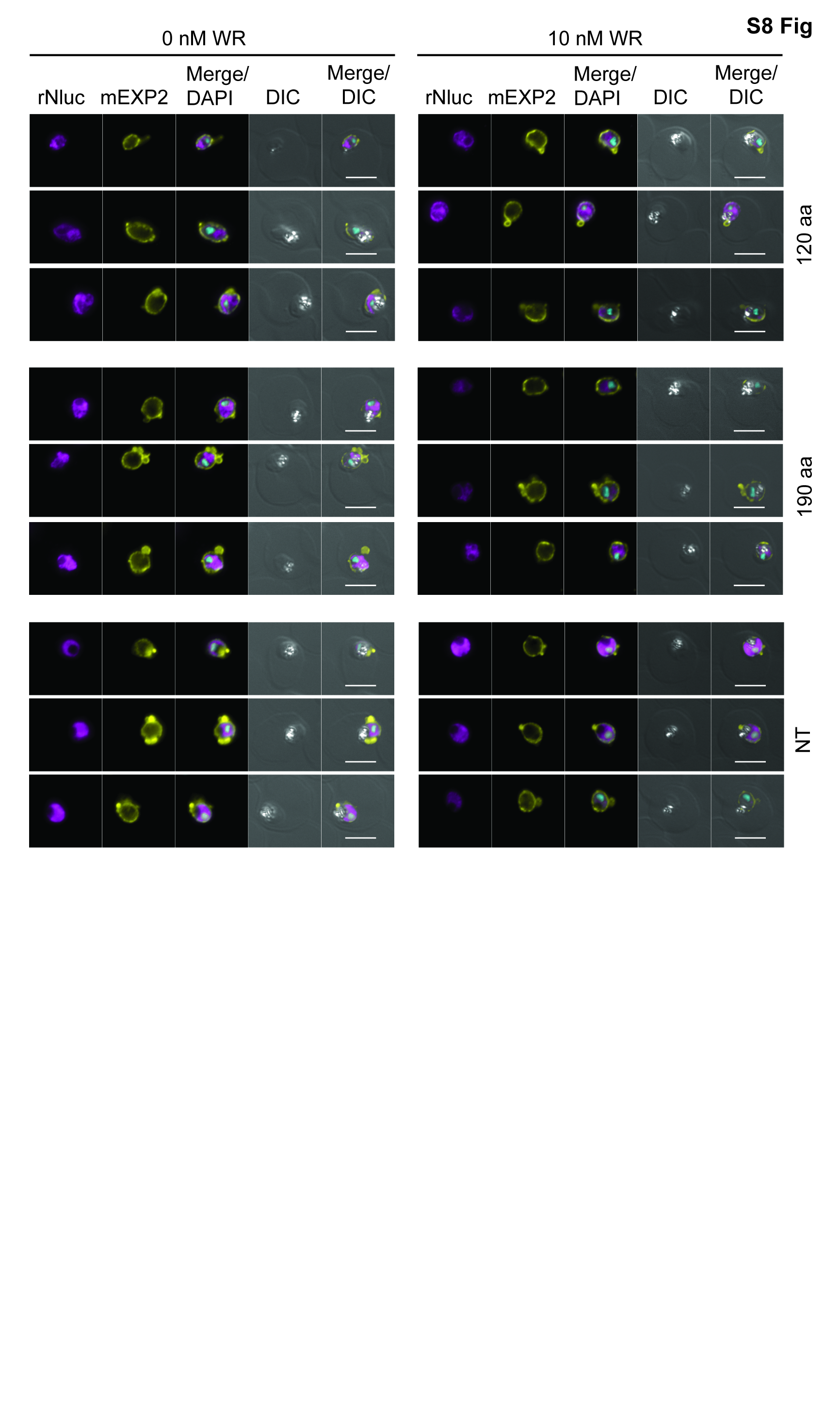

Supplement: S8 Fig — (TIF) [file ppat.1011006.s008.tif]

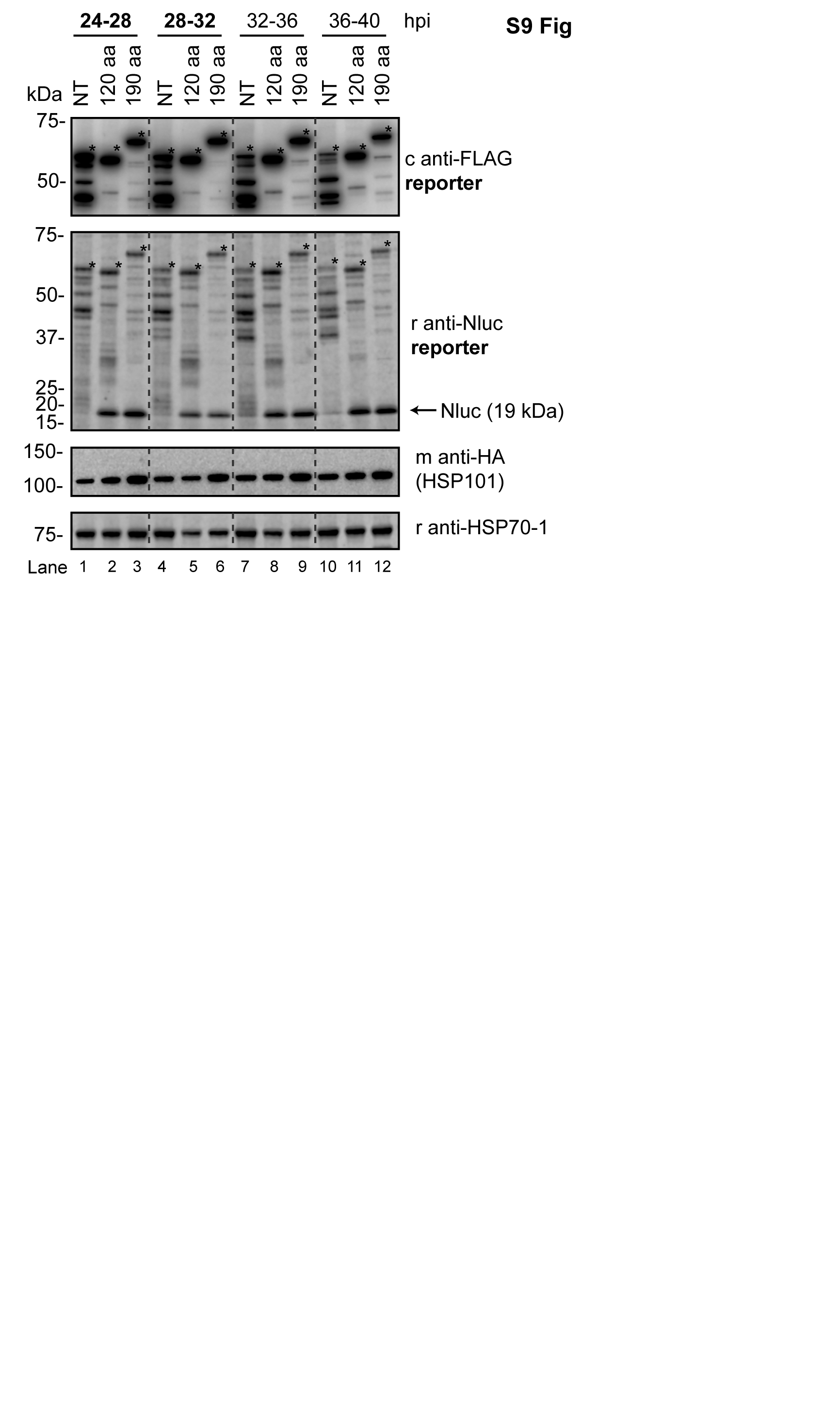

Supplement: S9 Fig — Synchronous (4-h invasion window) parasite cultures for the three FP2a reporter lines were divided in four and harvested via saponin lysis at four sequential time points: 24 to 28-hpi, 28 to 32-hpi, 32 to 36-hpi and 36 to 40-hpi. Chicken anti-FLAG and rabbit anti-Nluc were used to visualise the FP2a reporters, mouse anti-HA to visualise the HSP101-HAglmS (parental line) and rabbit anti-HSP70-1 was used as a loading control. The expected size of each reporter is indicated with (*), where the cleavage of Nluc from the 120 and 190 aa reporters was observed in each time point (lanes 2, 3, 5, 6, 8, 9, 11 and 12), likely due to cleavage upon entry into the food vacuole. This cleavage of Nluc was not observed for the NT reporter (lanes 1, 4, 7 and 10), which does not enter the food vacuole. The NT reporter was also degraded more, and the expression of the full-length reporter diminishes as the parasite matures (lanes 1 and 4 vs. lanes 7 and 10), whilst expression of the 120 and 190 aa reporters remains stable across each time point. These data indicate that the optimal time point to study these three reporters was in the range of 24 to 32-hpi, as indicated in bold. This blot represents 2 biological replicates. Full-length blots are shown in S17 Fig. (TIF) [file ppat.1011006.s009.tif]

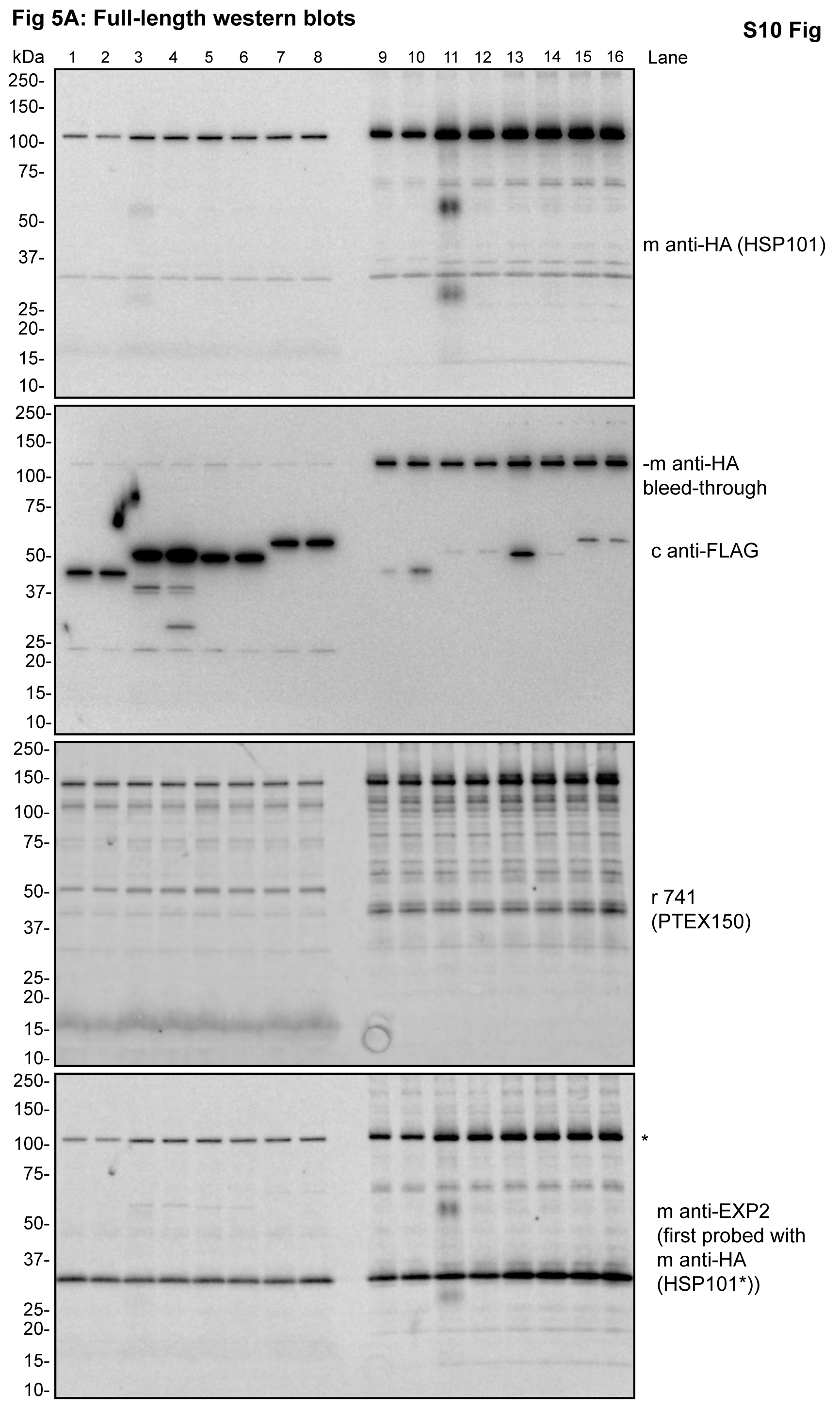

Supplement: S10 Fig — (TIF) [file ppat.1011006.s010.tif]

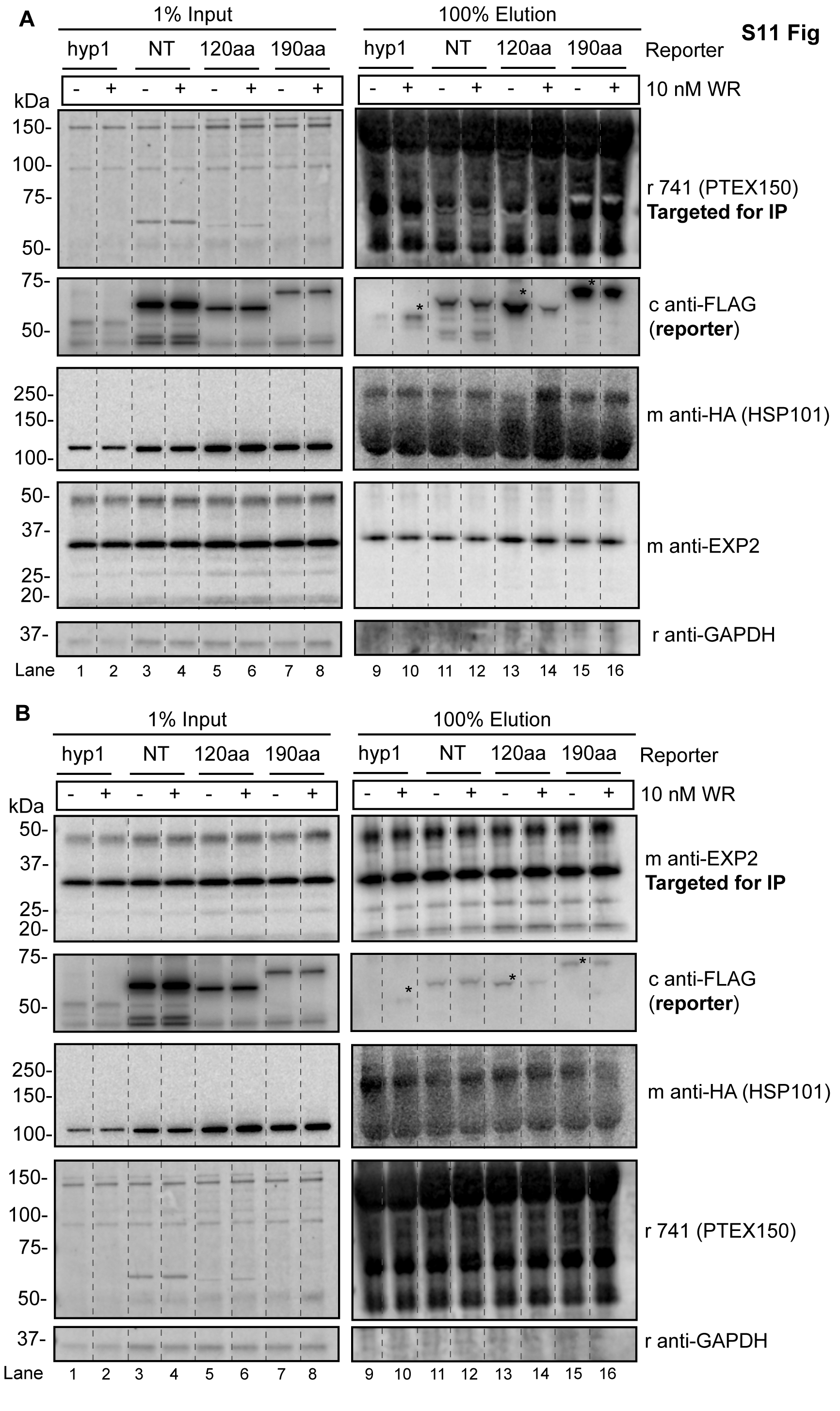

Supplement: S11 Fig — Immunoprecipitation assays with parasites treated with ± 10 nM WR99210 were completed on the four reporter lines using Protein Sepharose A where (A) PTEX150 or (B) EXP2 protein specific antibodies were incubated with parasite lysate to Co-IP interacting proteins. Both 120 aa (line 13) and 190 aa (line 15) showed stronger association with (A) PTEX150 and (B) EXP2 in–WR99210 treatment as determined by densitometry (S12 Fig). The Hyp1 reporter showed reverse association to FP2a reporters and NT showed some background signal in both conditions. (A) PTEX150 and (B) EXP2 both co-precipitated with other PTEX components (lanes 9–16). Chicken anti-FLAG was used to probe for the reporter, mouse anti-HA for HSP101-HAglmS and rabbit 741 for PTEX150. Both (A) and (B) blots represent 3 biological replicates. The asterisks indicate stronger signal when comparing the ± WR99210 treatment for respective reporter measured by densitometry of 3 biological replicates. Full-length blots are shown in S18–S21 Figs. (TIF) [file ppat.1011006.s011.tif]

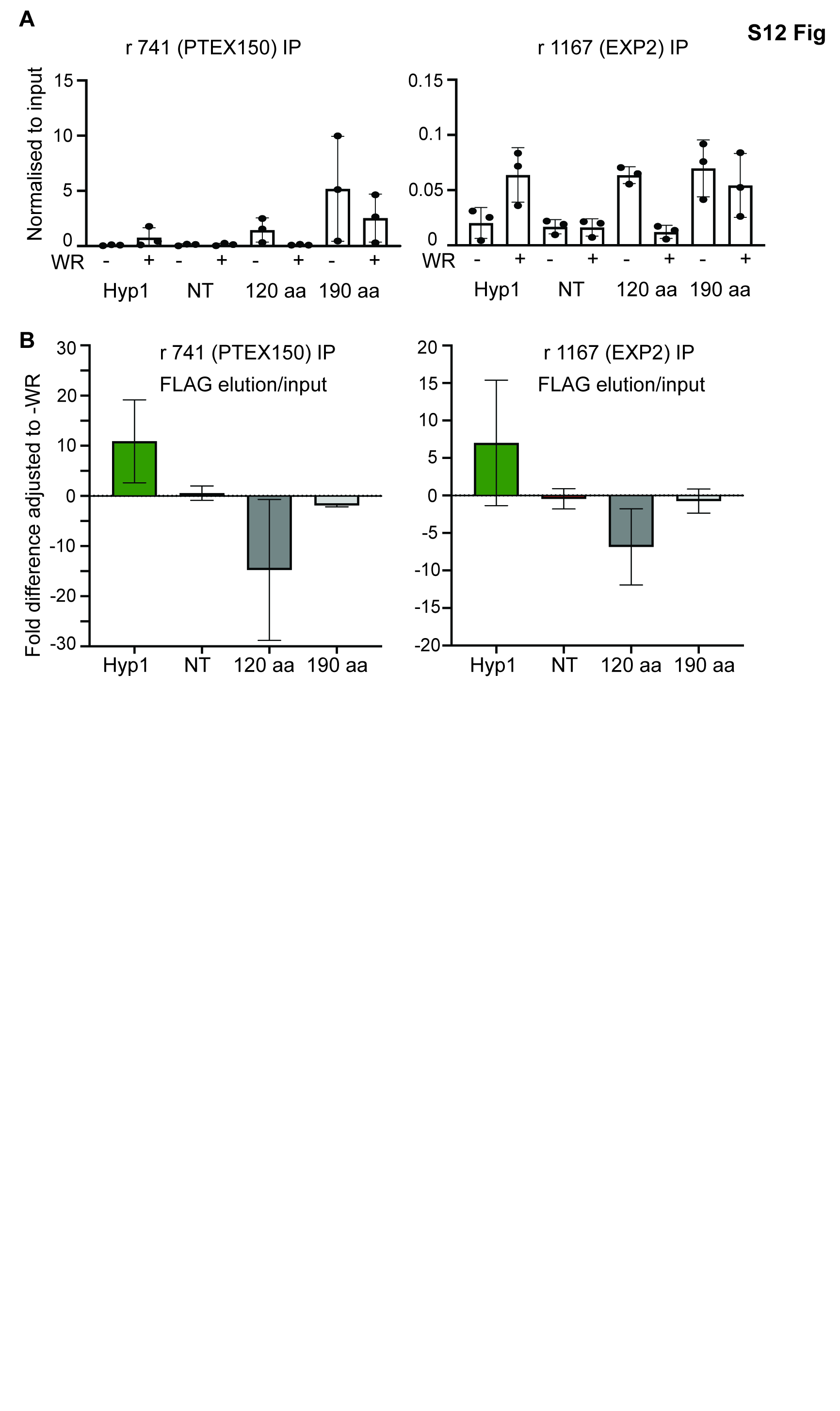

Supplement: S12 Fig — (A) The interaction of reporters with PTEX150 (r942) and EXP2 (r1167) presented in S11 Fig A was graphed, where FLAG elution was adjusted to input. Each dot represents 1 biological replicate. (B) The interaction of reporters with PTEX150 (r942) and EXP2 (r1167), where the fold difference of the FLAG elution/input was adjusted to untreated (- WR99210). Error bars = SD from 3 biological replicates. (TIF) [file ppat.1011006.s012.tif]

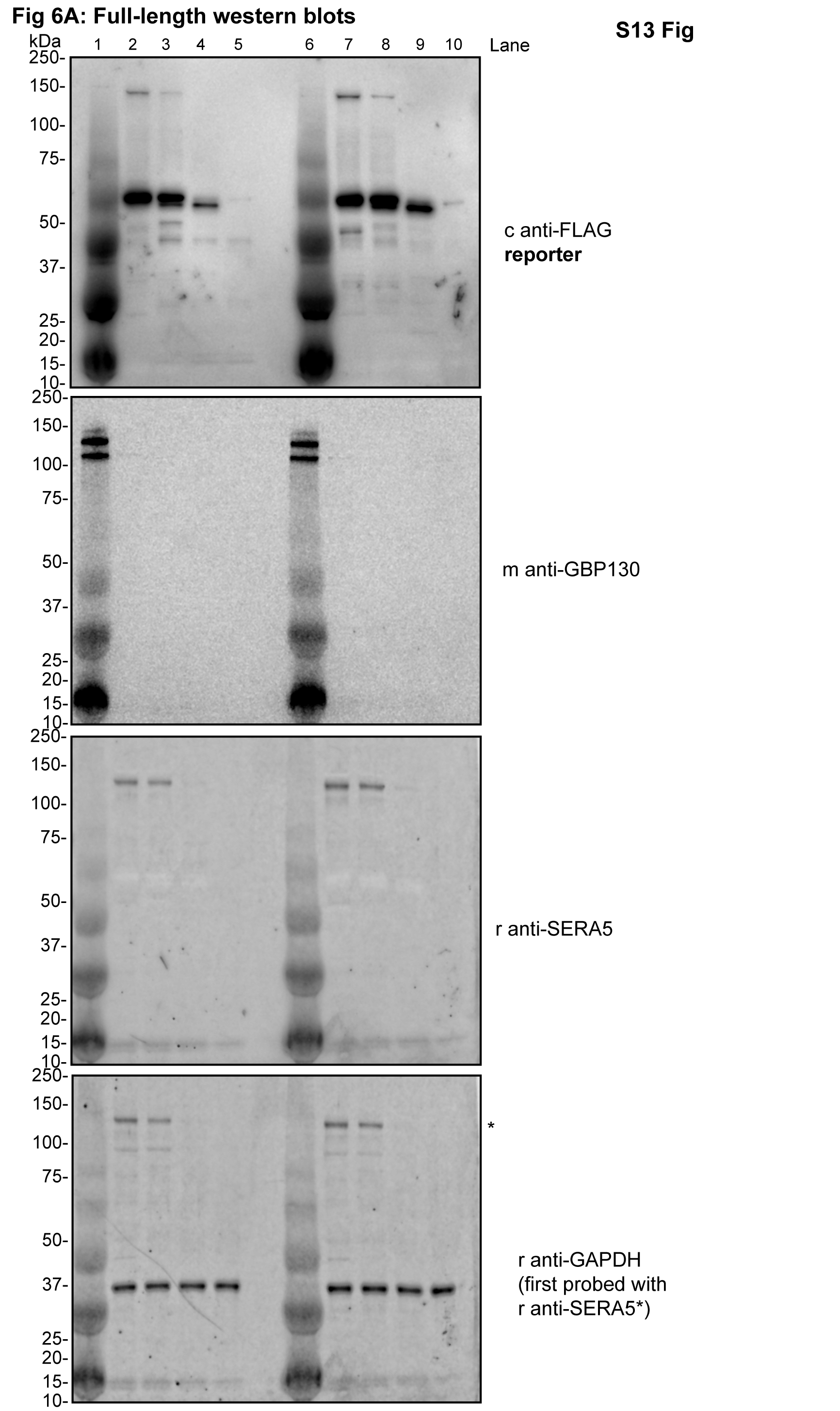

Supplement: S13 Fig — (TIF) [file ppat.1011006.s013.tif]

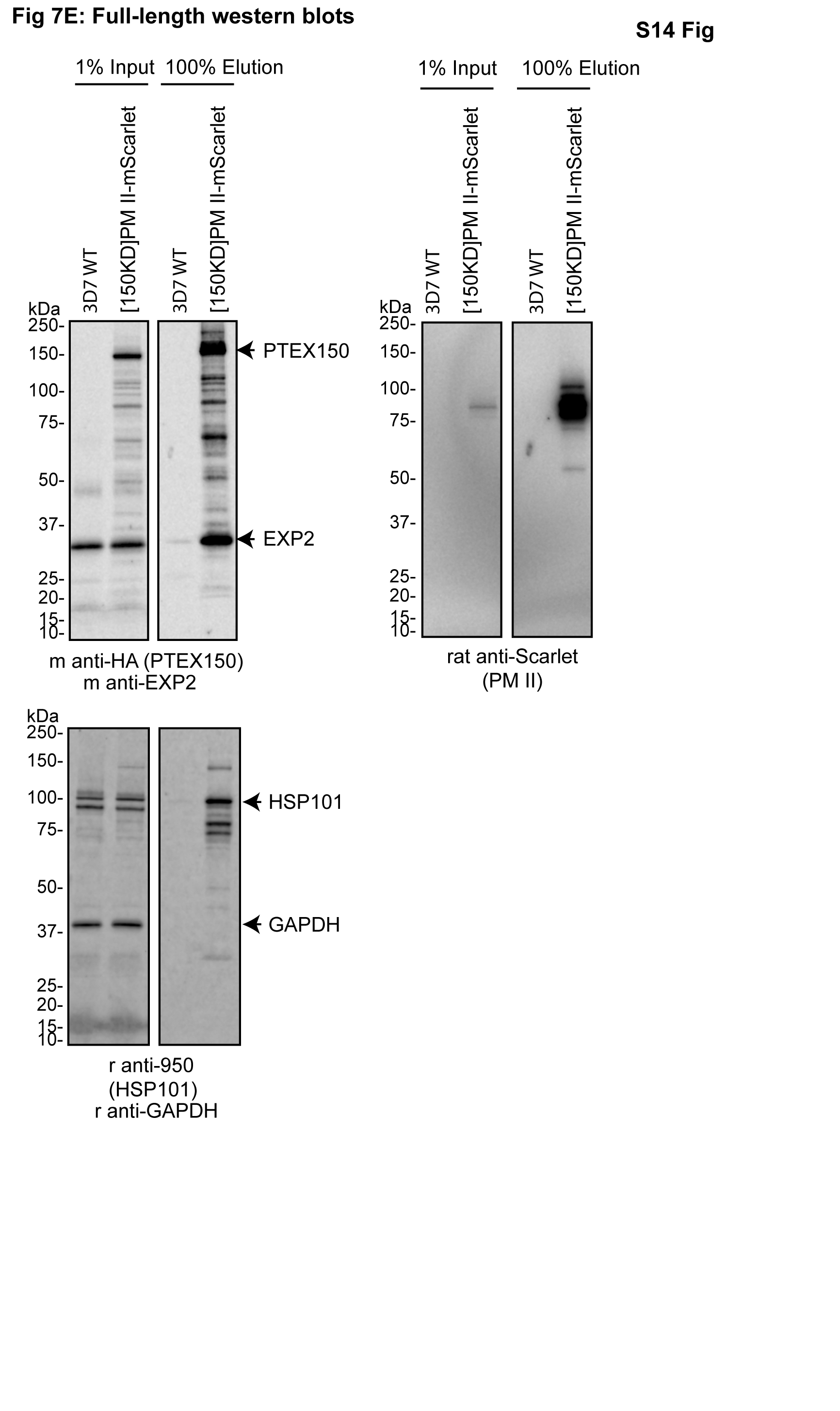

Supplement: S14 Fig — (TIF) [file ppat.1011006.s014.tif]

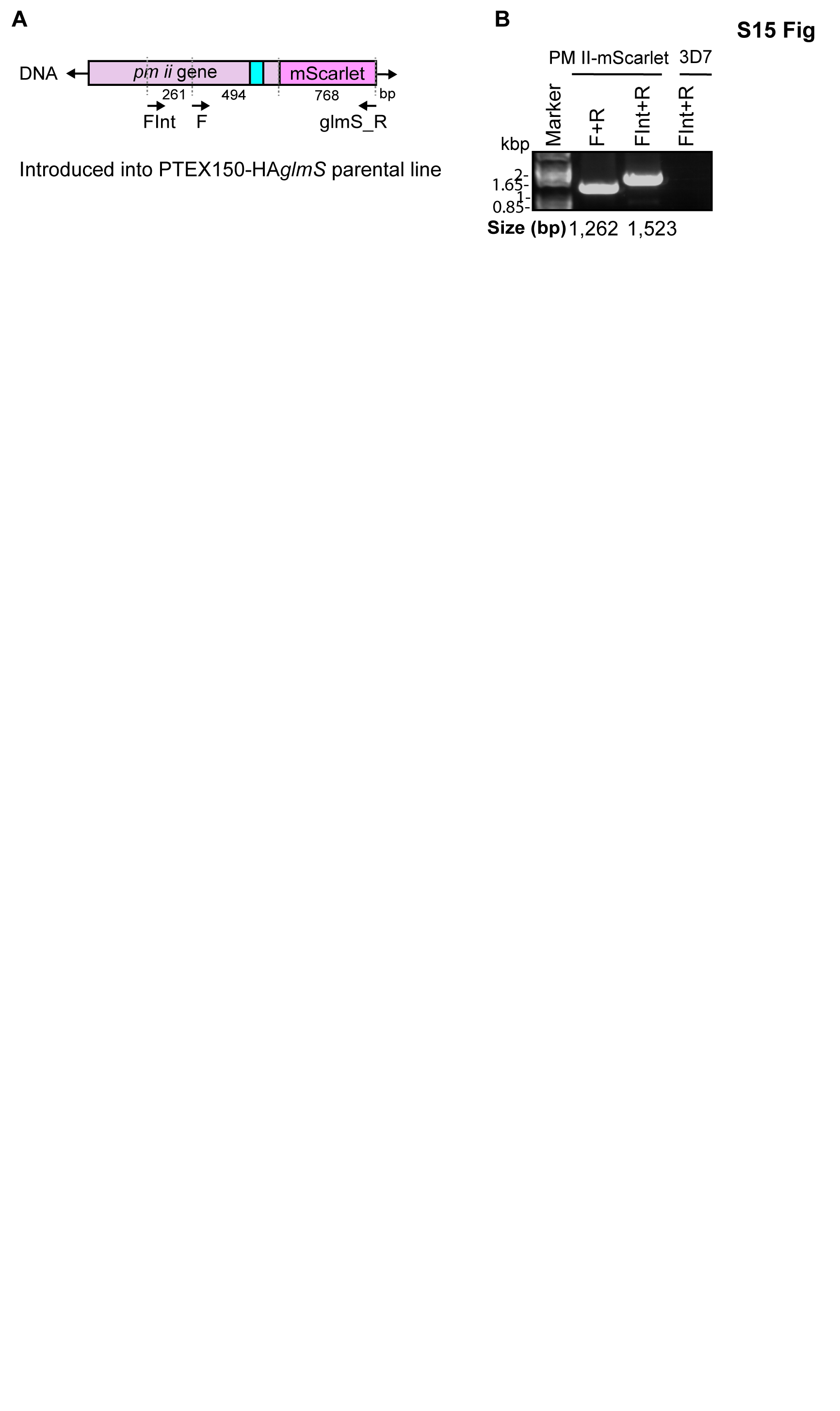

Supplement: S15 Fig — (A) The pm ii gene was C-terminally tagged with mScarlet as described for FP2a in S2 Fig A and introduced into a PTEX150-HAglmS background. The primers used to amplify the mScarlet tag were the same as for the HAglmS tag and all fragment joined together as explained for FP2a-HAglmS. (B) Correct integration of mScarlet to PM II was confirmed via PCR, where genotyping primers are displayed in panel A. (TIF) [file ppat.1011006.s015.tif]

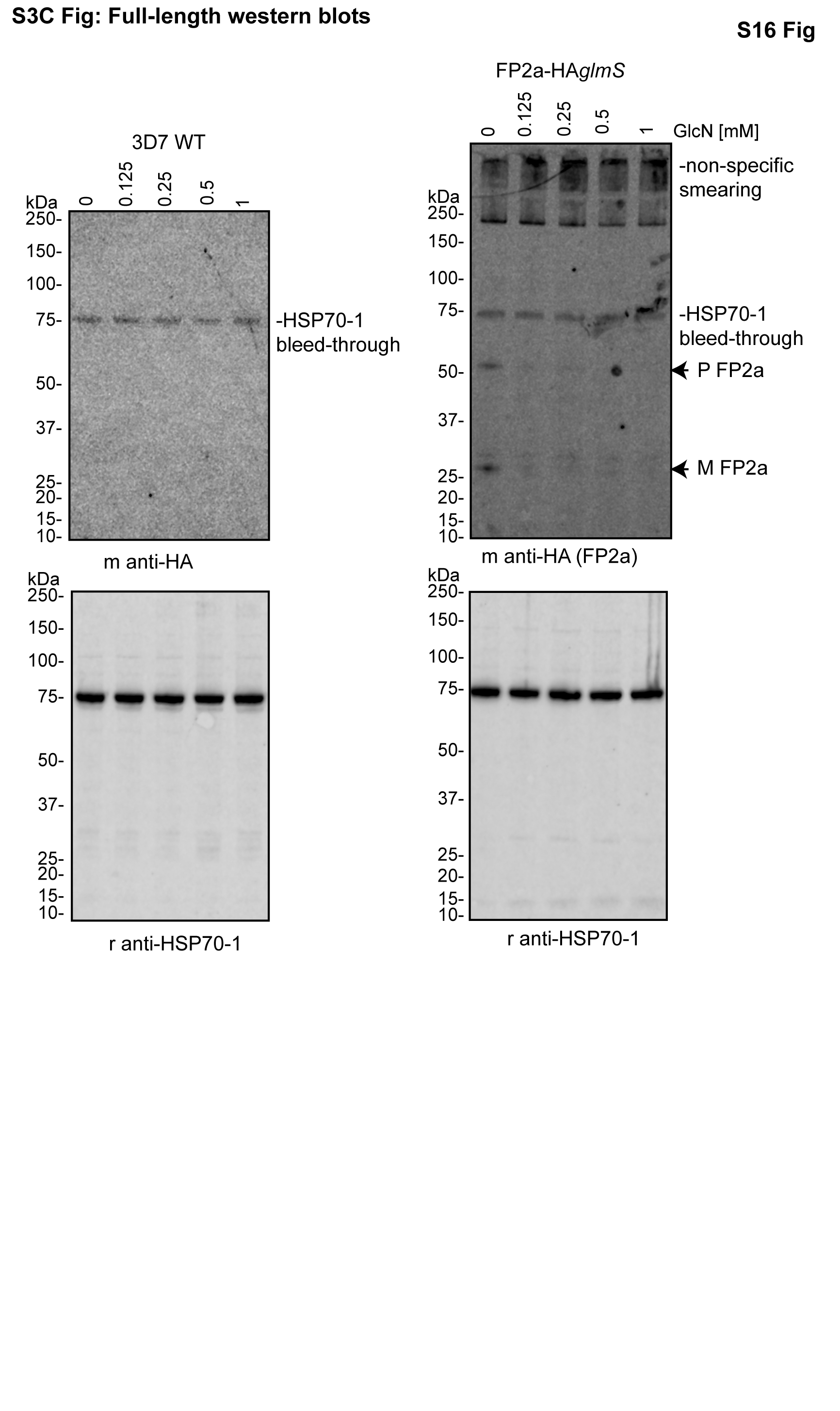

Supplement: S16 Fig — (TIF) [file ppat.1011006.s016.tif]

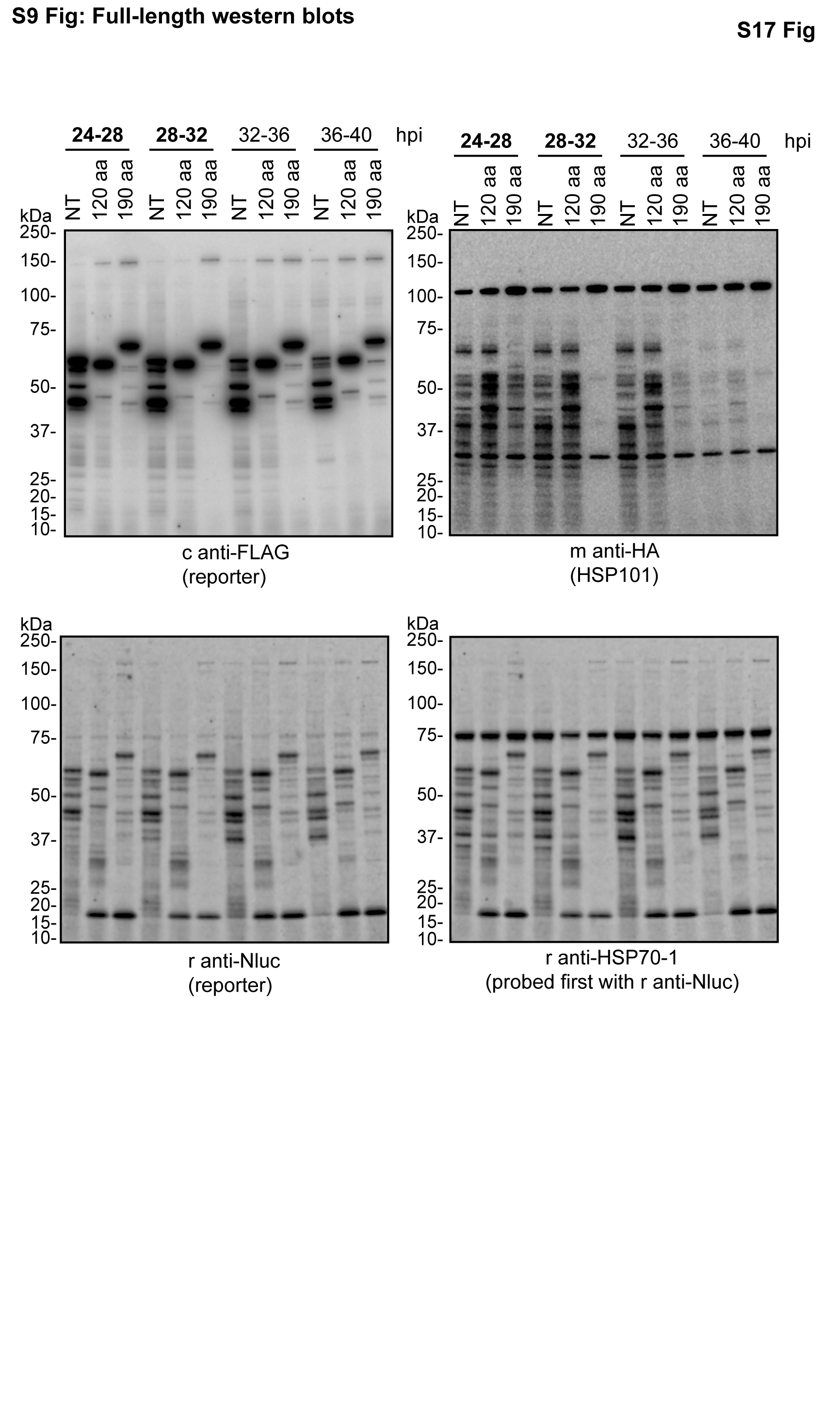

Supplement: S17 Fig — (TIF) [file ppat.1011006.s017.tif]

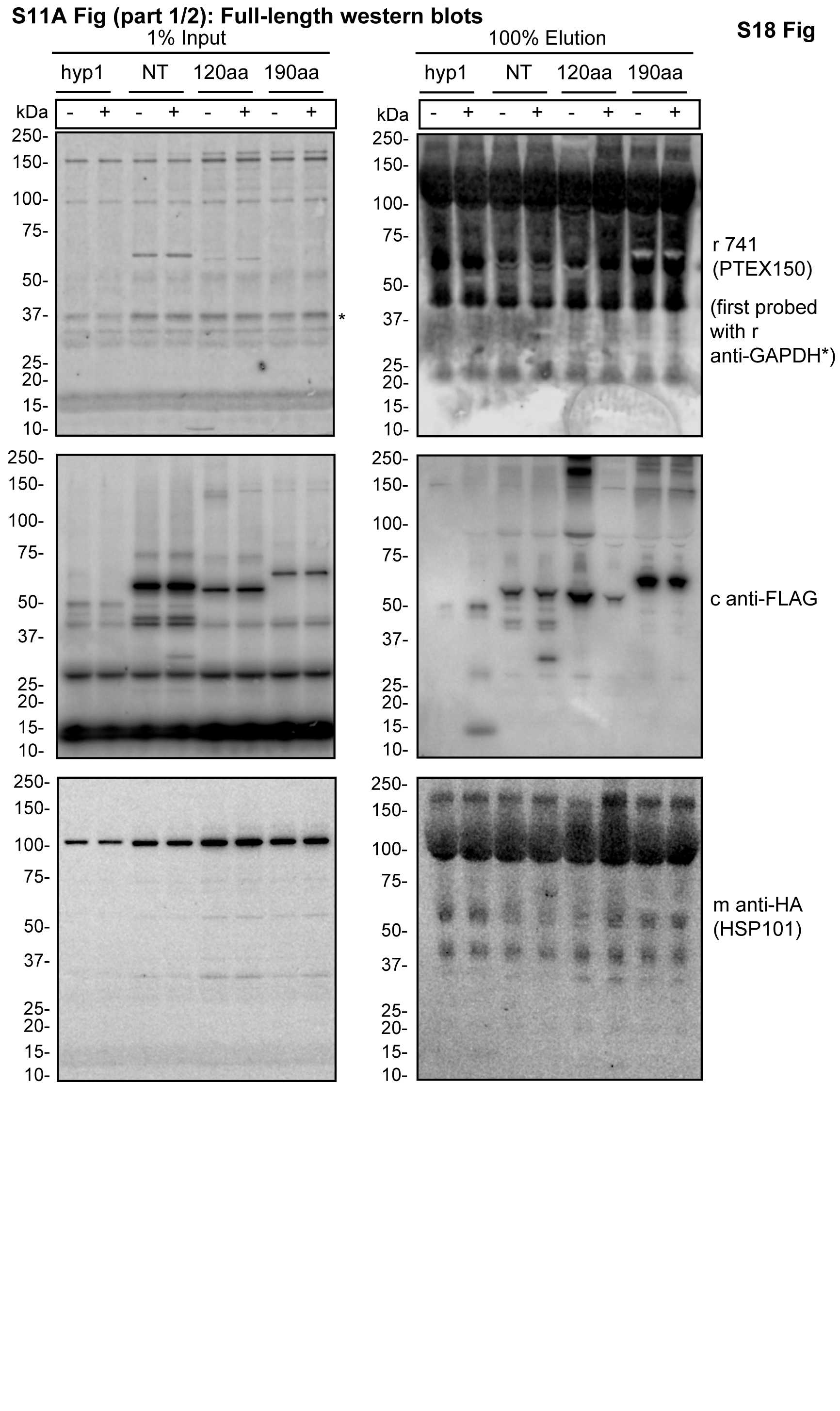

Supplement: S18 Fig — (TIF) [file ppat.1011006.s018.tif]

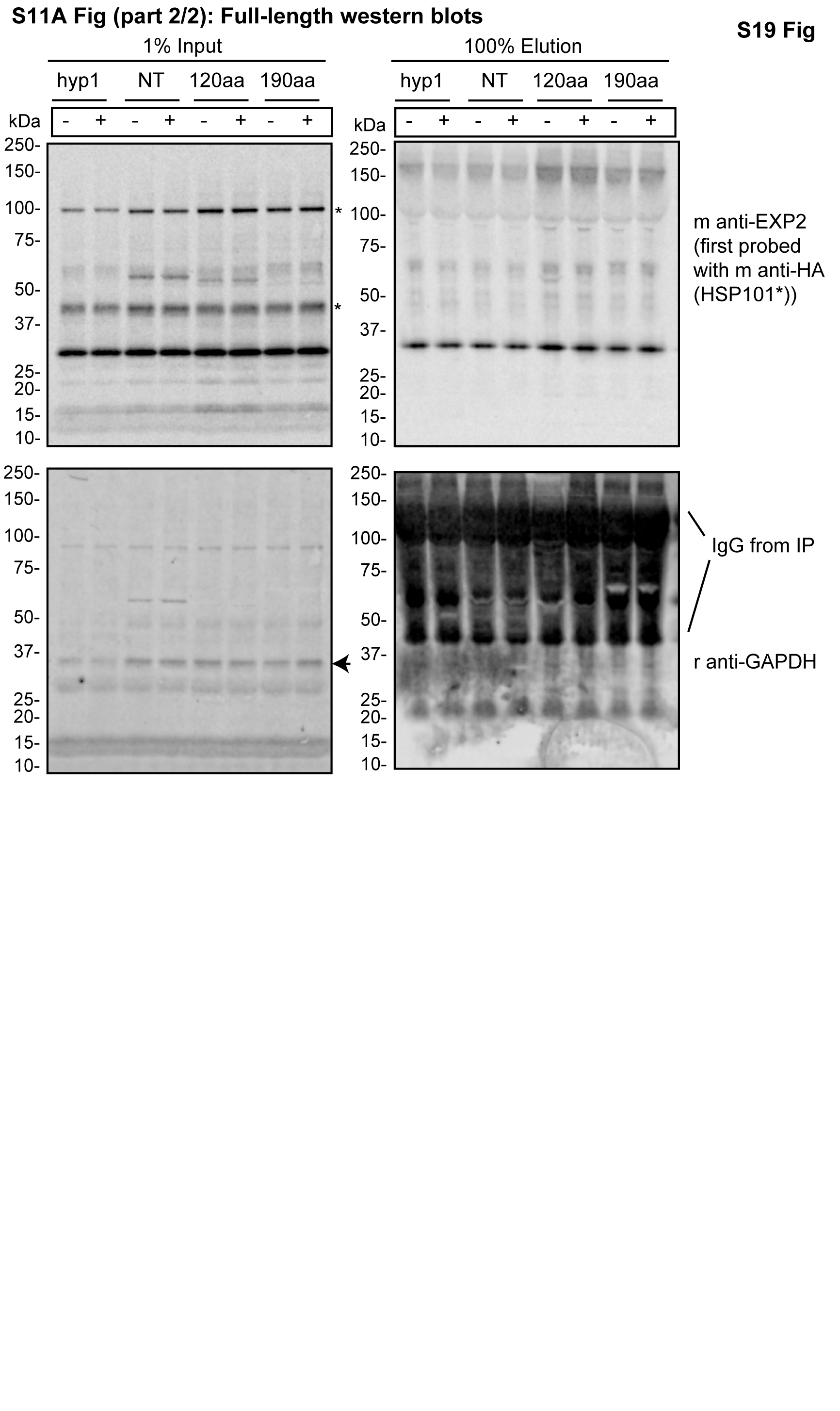

Supplement: S19 Fig — (TIF) [file ppat.1011006.s019.tif]

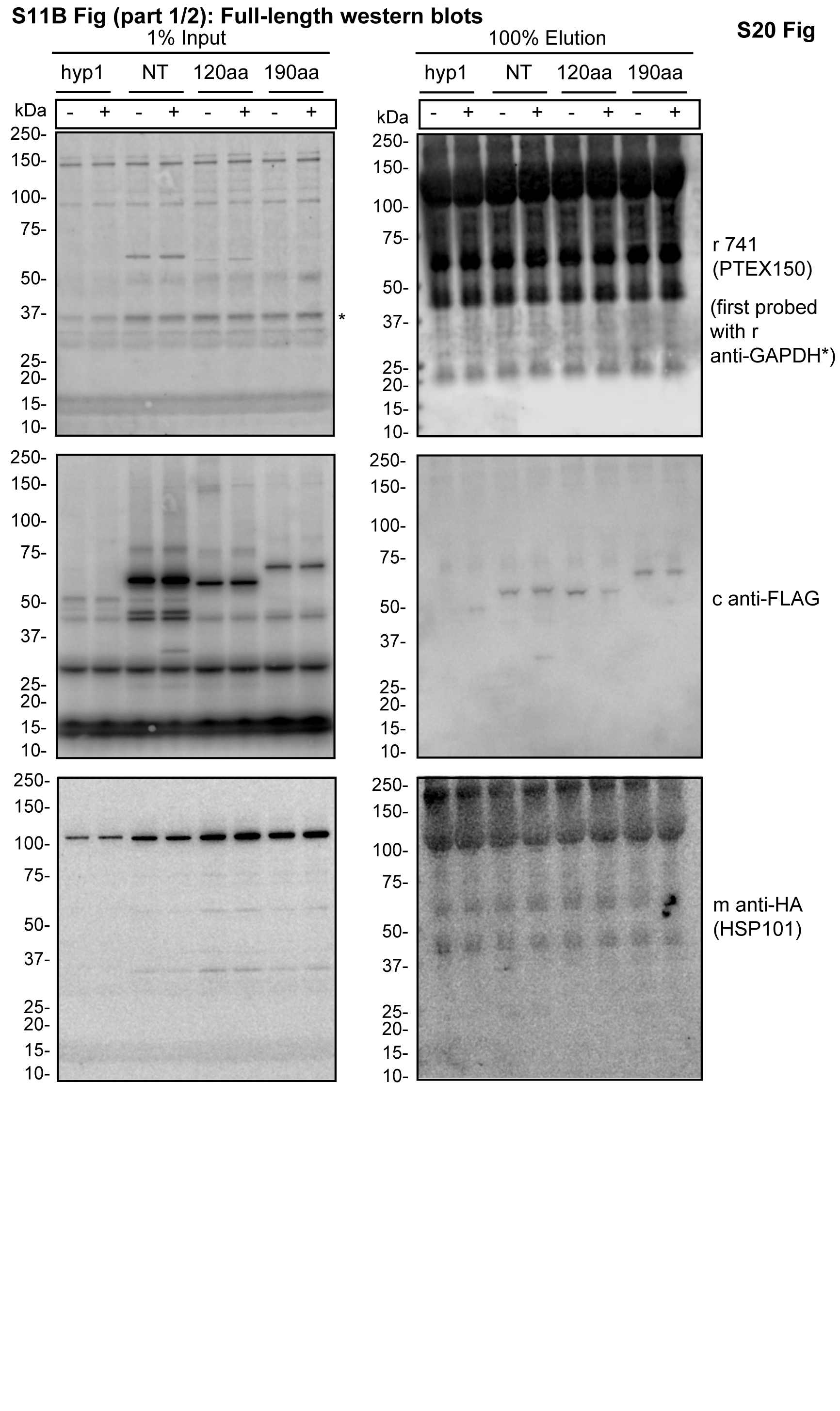

Supplement: S20 Fig — (TIF) [file ppat.1011006.s020.tif]

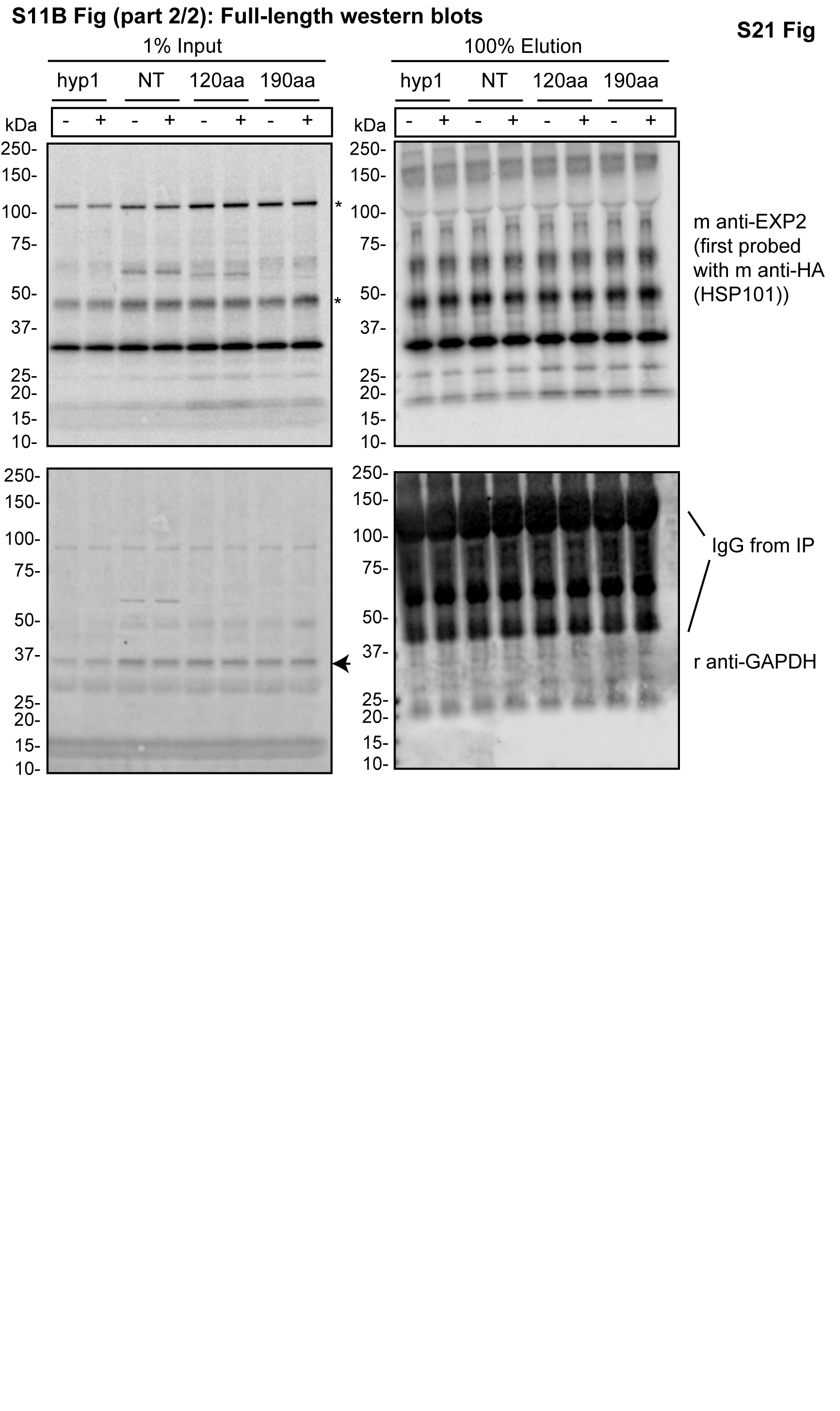

Supplement: S21 Fig — (TIF) [file ppat.1011006.s021.tif]
