# Supplementary material for: PTEX helps efficiently traffic haemoglobinases to the food vacuole in Plasmodium falciparum
Source: PLoS Pathog. 2023 Jul 31;19(7):e1011006. doi: 10.1371/journal.ppat.1011006 (PMC10414648; doi:10.1371/journal.ppat.1011006)
Supplement: S2 Table — (DOCX) [file ppat.1011006.s023.docx]

**S2 Table: List of primers and DNA sequences used in this study.**

| **List of primers** | |
| --- | --- |
| **PF3D7_1115700 (FP2a)** | |
| **FP2a-HA*glmS* line** | |
| 1F | TGATCATGCAGCTTATGATTGGAGATTAC |
| 2R | TCACCGCACTCACCGTCGAAAATACCTTCT |
| 3F | GGTATTTTCGACGGTGAGTGCGGTGATCAATTAAATCA |
| 4R | TCGTACGGGTAAGCTGCAGCTTCAATTAATGGAATGAATGCATCAGT |
| 5F | AAAGAGAAATCACATGATCTATATGTCAATAGTATTTTAAAAAGGGACCT |
| 6R | GTAAGGTAAAGGAAAAATTAGTAAGGATGCA |
| F_Int | CAAGTATTCTTACAAAATGCACACAAAG |
| sgRNA_F | TATTGGTATTTTCGATGGAGAATG |
| sgRNA_R | AAACCATTCTCCATCGAAAATACC |
| **Trappable FP2a constructs** | |
| *FP2a 120 aa* | |
| XhoF | **CTCGAG**CAAAATGGATTACAACATGGATTATGCT |
| NcoR | **CCATGG**TATTGTTGTTATTACCTTCGTTGTATTTATTTATGTCA |
| FP2a 190 aa | |
| XhoF | **CTCGAG**CAAAATGGATTACAACATGGATTATGCT |
| NcoR | **CCATGG**ATAAGAATACTTGAAATCTTTCCTTCATTTCATTTGGAGA |
| FP2a NT | |
| XhoF | **CTCGAG**CAAAATGACTCCAAATTCTAGAAAAAGTGA |
| NcoR | **CCATGG**ATAAGAATACTTGAAATCTTTCCTTCATTTCATTTGGAGA |
| **PF3D7_1408000 (PMII)** | |
| **PMII-mScarlet** | |
| 1F | ACAGGATTCTTAACCATTGGTGGT |
| 2R | GGGTCACCAAGGATGAATGTTGGTACTGGAAAATCTAATCCT |
| 3F | CCAGTACCAACATTCATCCTTGGTGACCCATTCATGA |
| 4R | CCCGCTGCGGCAGCTCCACTTAAATTCTTTTTAGCAAGAGCA |
| 5F | ATGTTCCAGATTATGCCTAAAAACGAAAATTTGGAAACCAAAGGA |
| 7F | ACCTTCAACACATAGAAGATGTTGGTCCAGGA |
| 6R | TTTTATGAACTCACTCTTGTGTTAGGT |
| F_Int | TGTCAGGAACTGTTAGTGGATTTTTCAGT |
| sgRNA_F | AAACACCTTTATTCTAGGTGACCC |
| sgRNA_R | TATTGGGTCACCTAGAATAAAGGT |
| **Protein tag primers** | |
| **HA*glmS*/mScarlet** | |
| F | GCTGCAGCTTACCCGTACGA |
| R | AGATCATGTGATTTCTCTTTGTTCA |
| **Replacing hDHFR with BSD in the pCas9 plasmid** | |
| BSD_NcoF | TTTCCATGGTTAAGCCTTTGTCTCAAGAAGAATCCA |
| BbsI_MutR | AGCAACGGCTACAATCAACAGCATCCCCA |
| BbsI_MutF | TGGGGATGCTGTTGATTGTAGCCGTTGCT |
| BSD_SacIIR | TAACCGCGGTTAGCCCTCCCACACATAACCA |
| **Protein tag sequences** | |
| 1XHA | TACCCGTACGACGTCCCGGACTACGCT |
| *glmS* | TAATTATAGCGCCCGAACTAAGCGCCCGGAAAAAGGCTTAGTTGACGAGGATGGAGGTTATCGAATTTTCGGCGGATGCCTCCCGGCTGAGTGTGCAGATCACAGCCGTAAGGATTTCTTCAAACCAAGGGGGTGACTCCTTGAACAAAGAGAAATCACATGATCT |
| mScarlet | GCTGCAGCTTACCCGTACGACGTCCCGGACTACGCTGGAGCAAGTATGGTGAGCAAGGGCGAGGCAGTGATCAAGGAGTTCATGCGGTTCAAGGTGCACATGGAGGGCTCCATGAACGGCCACGAGTTCGAGATCGAGGGCGAGGGCGAGGGCCGCCCCTACGAGGGCACCCAGACCGCCAAGCTGAAGGTGACCAAGGGTGGCCCCCTGCCCTTCTCCTGGGACATCCTGTCCCCTCAGTTCATGTACGGCTCCAGGGCCTTCATCAAGCACCCCGCCGACATCCCCGACTACTATAAGCAGTCCTTCCCCGAGGGCTTCAAGTGGGAGCGCGTGATGAACTTCGAGGACGGCGGCGCCGTGACCGTGACCCGGACACCTCCCTGGAGGACGGCACCCTGATCTACAAGGTGAAGCTCCGCGGCACCAACTTCCCTCCTGACGGCCCCGTAATGCAGAAGAAGACAATGGGCTGGGAAGCGTCCACCGAGCGGTTGTACCCCGAGGACGGCGTGCTGAAGGGCGACATTAAGATGGCCCTGCGCCTGAAGGACGGCGGCCGCTACCTGGCGGACTTCAAGACCACCTACAAGGCCAAGAAGCCCGTGCAGATGCCCGGCGCCTACAACGTCGACCGCAAGTTGGACATCACCTCCCACAACGAGGACTACACCGTGGTGGAACAGTACGAACGCTCCGAGGGCCGCCACTCCACCGGCGGCATGGACGAGCTGTACAAGTAATGAACAAAGAGAAATCACATGATCT |
| Nluc | TTCACACTCGAAGATTTCGTTGGGGACTGGCGACAGACAGCCGGCTACAACCTGGACCAAGTCCTTGAACAGGGAGGTGTGTCCAGTTTGTTTCAGAATCTCGGGGTGTCCGTAACTCCGATCCAAAGGATTGTCCTGAGCGGTGAAAATGGGCTGAAGATCGACATCCATGTCATCATCCCGTATGAAGGTCTGAGCGGCGACCAAATGGGCCAGATCGAAAAAATTTTTAAGGTGGTGTACCCTGTGGATGATCATCACTTTAAGGTGATCCTGCACTATGGCACACTGGTAATCGACGGGGTTACGCCGAACATGATCGACTATTTCGGACGGCCGTATGAAGGCATCGCCGTGTTCGACGGCAAAAAGATCACTGTAACAGGGACCCTGTGGAACGGCAACAAAATTATCGACGAGCGCCTGATCAACCCCGACGGCTCCCTGCTGTTCCGAGTAACCATCAACGGAGTGACCGGCTGGCGGCTGTGCGAACGCATTCTGGCG |
| DH | ATGGCGACCATTGAACTGCATCGTCGCCGTGTCCCAAAATATGGGGATTGGCAAGAACGGAGACCTACCCTGGCCTCCGCTCAGGAACGAGTTCAAGTACTTCCAAAGAATGACCACAACCTCTTCAGTGGAAGGTAAACAGAATCTGGTGATTATGGGTAGGAAAACCTGGTTCTCCATTCCTGAGAAGAATCGACCTTTAAAGGACAGAATTAATATAGTTCTCAGTAGAGAACTCAAAGAACCACCACGAGGAGCTCATTTTCTTGCCAAAAGTTTGGATGATGCGTTAAGACTTATTGAACAACCGGAATTGGCAAGTAAAGTAGACATGGTTTGGATAGTCGGAGGCAGTTCTGTTTACCAGGAAGCCATGAATCAACCAGGCCACCTCAGACTCTTTGTGACAAGGATCATGCAGGAATTTGAAAGTGACACGTTTTTCCCAGAAATTGATTTGGGGAAATATAAACTTCTCCCAGAATACCCAGGCGTCCTCTCTGAGGTCCAGGAGGAAAAAGGCATCAAGTATAAGTTTGAAGTCTACGAGAAGAAAGACATA |
| 3XFLAG | GATTATAAAGACGATGACGATAAGGACTACAAGGATGACGATGACAAAGATTACAAAGATGATGATGATAAA |

F refers to forward primers and R to reverse primers.
