# Supplementary material for: PTEX helps efficiently traffic haemoglobinases to the food vacuole in Plasmodium falciparum
Source: PLoS Pathog. 2023 Jul 31;19(7):e1011006. doi: 10.1371/journal.ppat.1011006 (PMC10414648; doi:10.1371/journal.ppat.1011006)
Supplement: S3 Table — (DOCX) [file ppat.1011006.s024.docx]

**S3 Table: List of antibodies used**

| **Antibody** | **Species** | **Dilution / Concentration** | | | **Reference** |
| --- | --- | --- | --- | --- | --- |
|  |  | *IFA* | *WB* | *IP* |  |
| Anti-HA | Mouse | 1:1,000 | 1:1,000 |  | Sigma-Aldrich, ref. H3663-200UL (knockdown blots) |
| Anti-HA | Mouse |  | 1:500 |  | WEHI monoclonal antibody facility (IP blots) |
| Anti-FLAG | Chicken |  | 1:2,000 |  | Abcam, ref. ab1170 |
| Anti-FLAG | Mouse | 1:1,000 |  |  | Sigma-Aldrich, ref. F1804-50UG |
| Anti-RFP | Rat |  | 1:1,000 |  | Chromotek, ref. 5F8-20 |
| Anti-Nanoluciferase IgG purified | Rabbit | 1:300 | 1:300 |  | WEHI monoclonal antibody facility |
| Anti-EXP2 IgG purified | Mouse | 5 µg/ml | 5 µg/ml |  | WEHI monoclonal antibody facility |
| Anti-EXP2 (R1167) | Rabbit | 1:1,000 | 1:1,000 – 1:2,000 | 20 µl | WEHI monoclonal antibody facility |
| Anti-PTEX150 (741) | Rabbit |  | 1:500 |  | WEHI monoclonal antibody facility |
| Anti-PTEX150 (942) | Rabbit |  |  | 20µl | WEHI monoclonal antibody facility |
| Anti-HSP70-1 | Rabbit |  | 1:1,000 |  | WEHI monoclonal antibody facility |
| Anti-GAPDH | Rabbit |  | 1:2,000 |  | Kind gift from Leann Tilley and Matthew Dixon |
| Anti-GBP130 | Mouse |  | 1:1,000 |  | WEHI monoclonal antibody facility |
| Anti-SERA5 | Rabbit |  | 1:1,000 |  | WEHI monoclonal antibody facility |
| Anti-CRT | Rabbit | 1:300 |  |  | Kind gift from Leann Tilley and Matthew Dixon |
| Anti-ERC | Rabbit | 1:1,000 |  |  | Kind gift from Leann Tilley and Matthew Dixon |
| Anti-Human Haemoblogin | Rabbit | 1:1,000 | 1:1,000 |  | WEHI monoclonal antibody facility |
| Anti-Mouse IgG Alexa Fluor Plus 488 | Goat | 1:2,000 |  |  | Invitrogen |
| Anti-Rabbit IgG Alexa Fluor Plus 594 | Goat | 1:2,000 |  |  | Invitrogen |
| Anti-Mouse IgG Alexa Fluor Plus 594 | Goat | 1:2,000 |  |  | Invitrogen |
| Anti-Rabbit IgG Alexa Fluor Plus 700 | Goat |  | 1:10,000 |  | Invitrogen |
| Anti-Mouse IgG Alexa Fluor Plus 800 | Goat |  | 1:10,000 |  | Invitrogen |
| Anti-Chicken IgY (HRP) | Goat |  | 1:10,000 |  | Abcam |
| Anti-Rat IgG (HRP) | Rabbit |  | 1:10,000 |  | Dako |
